# 2-Amino-5-methyl­pyridinium 4-hydroxy­benzoate

**DOI:** 10.1107/S1600536810009396

**Published:** 2010-03-27

**Authors:** Madhukar Hemamalini, Hoong-Kun Fun

**Affiliations:** a X-ray Crystallography Unit, School of Physics, Universiti Sains Malaysia, 11800 USM, Penang, Malaysia

## Abstract

In the title salt, C_6_H_9_N_2_^+^·C_7_H_5_O_3_^−^, the carboxyl­ate mean plane of the 4-hydroxy­benzoate anion is twisted by 13.07 (4)° from the attached ring. In the crystal structure, the ions are linked into a two-dimensional network by N—H⋯O, O—H⋯O and C—H⋯O hydrogen bonds. Within this network, the N—H⋯O hydrogen bonds generate *R*_2_^2^(8) ring motifs. In addition, π–π inter­actions involving the pyridinium rings, with a centroid–centroid distance of 3.7599 (4) Å, are observed.

## Related literature

For background to the chemistry of substituted pyridines, see: Pozharski *et al.* (1997 ▶); Katritzky *et al.* (1996 ▶). For related structures, see: Hemamalini & Fun (2010*a* ▶,*b* ▶,*c* ▶). For 4-hydroxy­benzoic acid, see: Vishweshwar *et al.* (2003 ▶). For details of hydrogen bonding, see: Jeffrey & Saenger (1991 ▶); Jeffrey (1997 ▶); Scheiner (1997 ▶); Aakeröy *et al.* (2002 ▶). For hydrogen-bond motifs, see: Bernstein *et al.* (1995 ▶). For bond-length data, see: Allen *et al.* (1987 ▶). For the stability of the temperature controller used in the data collection, see: Cosier & Glazer (1986 ▶).
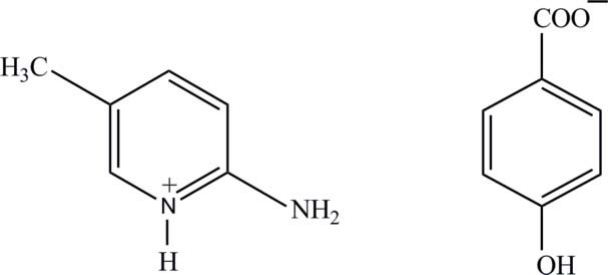


## Experimental

### 

#### Crystal data

C_6_H_9_N_2_^+^·C_7_H_5_O_3_^−^
*M* *_r_* = 246.26
Monoclinic, 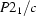

*a* = 12.9562 (6) Å
*b* = 8.7876 (4) Å
*c* = 11.3276 (5) Å
β = 108.397 (1)°
*V* = 1223.78 (10) Å^3^
*Z* = 4
Mo *K*α radiation
μ = 0.10 mm^−1^
*T* = 100 K
0.39 × 0.33 × 0.27 mm

#### Data collection

Bruker APEX DUO CCD area-detector diffractometer
Absorption correction: multi-scan (*SADABS*; Bruker, 2009 ▶) *T*_min_ = 0.963, *T*_max_ = 0.975
20102 measured reflections
5326 independent reflections
4662 reflections with *I* > 2σ(*I*)
*R*_int_ = 0.019

#### Refinement

*R*[*F* ^2^ > 2σ(*F* ^2^)] = 0.039
*wR*(*F* ^2^) = 0.137
*S* = 1.15
5326 reflections
219 parameters
H atoms treated by a mixture of independent and constrained refinement
Δρ_max_ = 0.60 e Å^−3^
Δρ_min_ = −0.32 e Å^−3^

### 

Data collection: *APEX2* (Bruker, 2009 ▶); cell refinement: *SAINT* (Bruker, 2009 ▶); data reduction: *SAINT*; program(s) used to solve structure: *SHELXTL* (Sheldrick, 2008 ▶); program(s) used to refine structure: *SHELXTL*; molecular graphics: *SHELXTL*; software used to prepare material for publication: *SHELXTL* and *PLATON* (Spek, 2009 ▶).

## Supplementary Material

Crystal structure: contains datablocks global, I. DOI: 10.1107/S1600536810009396/rz2424sup1.cif

Structure factors: contains datablocks I. DOI: 10.1107/S1600536810009396/rz2424Isup2.hkl

Additional supplementary materials:  crystallographic information; 3D view; checkCIF report

## Figures and Tables

**Table 1:** Hydrogen-bond geometry (Å, °)

*D*—H⋯*A*	*D*—H	H⋯*A*	*D*⋯*A*	*D*—H⋯*A*
N1—H1*N*1⋯O3	0.957 (17)	1.726 (17)	2.6738 (9)	170.4 (15)
N2—H1*N*2⋯O3^i^	0.905 (14)	1.967 (14)	2.8443 (9)	162.9 (13)
N2—H2*N*2⋯O2	0.922 (14)	1.876 (14)	2.7962 (9)	176.1 (13)
O1—H101⋯O2^ii^	0.892 (18)	1.779 (18)	2.6635 (8)	170.7 (19)
C3—H3*A*⋯O2^iii^	1.016 (16)	2.476 (15)	3.1887 (9)	126.7 (10)

Symmetry codes: (i) 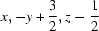; (ii) 
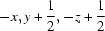
; (iii) 
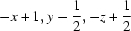
.

**Fig. 1.**
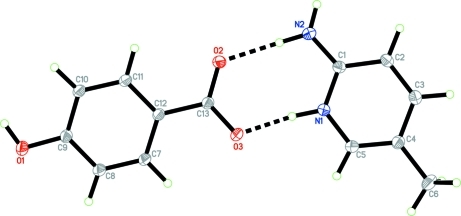
The asymmetric unit of the title compound. Displacement ellipsoids are drawn at the 50% probability level.

**Fig. 2.**
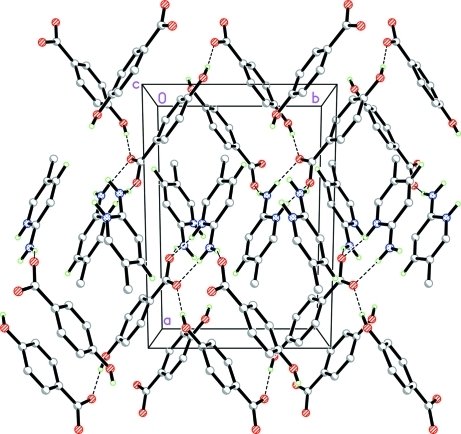
The crystal packing of the title compound, showing hydrogen-bonded (dashed lines) networks. H atoms are not involing the hydrogen bond interactions are omitted for clarity.

## supplementary crystallographic information

### Comment

In recent years, hydrogen bonds have attracted the interest of chemists and have been widely used to design and synthesize one, two and three-dimensional supramolecular compounds (Aakeröy *et al.*, 2002). Pyridine and its derivatives play an important role in heterocyclic chemistry (Pozharski *et al.*, 1997; Katritzky *et al.*, 1996). They are often involved in hydrogen-bond interactions (Jeffrey & Saenger, 1991; Jeffrey, 1997; Scheiner, 1997). 4-Hydroxybenzoic acid is a good hydrogen-bond donor and can form cocrystals with other organic molecules (Vishweshwar *et al.*, 2003). We have recently reported the crystal structures of 2-amino-5-methylpyridinium 3-aminobenzoate (Hemamalini & Fun, 2010a), 2-amino-5-methylpyridinium 4-nitrobenzoate (Hemamalini & Fun, 2010b) and 2-amino-5-methylpyridinium nicotinate (Hemamalini & Fun, 2010c). In continuation of our studies of pyrimidinium derivatives, the crystal structure determination of the title compound has been undertaken.

The asymmetric unit (Fig. 1) contains a 2-amino-5-methylpyridinium cation and a 4-hydroxybenzoate anion. In the 2-amino-5-methylpyridinium cation, a wide angle (122.65 (6)°) is subtended at the protonated N1 atom. The 2-amino-5-methylpyridinium cation is planar, with a maximum deviation of 0.024 (1)Å for atom N1. The bond lengths are normal (Allen *et al.*, 1987). In the 4-hydroxybenzoate anion, the carboxylate group is twisted slightly from the attached ring; the dihedral angle between C7–C12 and O2/O3/C12–C13 planes is 13.07 (4)°.

In the crystal packing (Fig. 2), the protonated N1 atom and the 2-amino group N2 atom are hydrogen-bonded to the carboxylate oxygen atoms (O2 and O3) via a pair of N—H···O hydrogen bonds forming a ring motif *R*_2_^2^(8) (Bernstein *et al.*, 1995).  The hydroxyl group hydrogen atom is also hydrogen-bonded to the carboxylate oxygen atom through O1—H1O1···O2 hydrogen bonds.  The packing is further stabilized by weak C—H···O and π–π interactions involving the pyridinium (centroid Cg1) rings, with Cg1–Cg1 = 3.7599 (4)Å [symmetry codes: 1-x, 1-y, 1-z].

### Experimental

A hot methanol solution (20 ml) of 2-amino-5-methylpyridine (54 mg, Aldrich) and 4-hydroxybenzoic acid (69 mg, Merck) were mixed and warmed over a heating magnetic stirrer for a few minutes.  The resulting solution was allowed to cool slowly at room temperature and crystals of the title compound appeared after a few days.

### Refinement

All the H atoms were located in a difference Fourier map and allowed to refine freely [N—H = 0.904 (14) - 0.956 (16)Å, C—H = 0.952 (16) - 1.020 (15)Å and O—H = 0.893 (18)Å].

### Crystal data

**Table:**

C_6_H_9_N_2_^+^·C_7_H_5_O_3_^−^	*F*(000) = 520
*M**_r_* = 246.26	*D*_x_ = 1.337 Mg m^−^^3^
Monoclinic, *P*2_1_/*c*	Mo *K*α radiation, λ = 0.71073 Å
Hall symbol: -P 2ybc	Cell parameters from 9907 reflections
*a* = 12.9562 (6) Å	θ = 6.5–36.2°
*b* = 8.7876 (4) Å	µ = 0.10 mm^−^^1^
*c* = 11.3276 (5) Å	*T* = 100 K
β = 108.397 (1)°	Blcok, colourless
*V* = 1223.78 (10) Å^3^	0.39 × 0.33 × 0.27 mm
*Z* = 4	

### Data collection

**Table:**

Bruker APEX DUO CCD area-detector diffractometer	5326 independent reflections
Radiation source: fine-focus sealed tube	4662 reflections with *I* > 2σ(*I*)
graphite	*R*_int_ = 0.019
φ and ω scans	θ_max_ = 35.0°, θ_min_ = 6.2°
Absorption correction: multi-scan (*SADABS*; Bruker, 2009)	*h* = −19→20
*T*_min_ = 0.963, *T*_max_ = 0.975	*k* = −14→11
20102 measured reflections	*l* = −18→15

### Refinement

**Table:**

Refinement on *F*^2^	Primary atom site location: structure-invariant direct methods
Least-squares matrix: full	Secondary atom site location: difference Fourier map
*R*[*F*^2^ > 2σ(*F*^2^)] = 0.039	Hydrogen site location: inferred from neighbouring sites
*wR*(*F*^2^) = 0.137	H atoms treated by a mixture of independent and constrained refinement
*S* = 1.15	*w* = 1/[σ^2^(*F*_o_^2^) + (0.0859*P*)^2^ + 0.1288*P*] where *P* = (*F*_o_^2^ + 2*F*_c_^2^)/3
5326 reflections	(Δ/σ)_max_ = 0.001
219 parameters	Δρ_max_ = 0.60 e Å^−^^3^
0 restraints	Δρ_min_ = −0.32 e Å^−^^3^

### Special details

**Table:**

Experimental. The crystal was placed in the cold stream of an Oxford Cryosystems Cobra open-flow nitrogen cryostat (Cosier & Glazer, 1986) operating at 100.0 (1) K.
Geometry. All s.u.'s (except the s.u. in the dihedral angle between two l.s. planes) are estimated using the full covariance matrix. The cell s.u.'s are taken into account individually in the estimation of s.u.'s in distances, angles and torsion angles; correlations between s.u.'s in cell parameters are only used when they are defined by crystal symmetry. An approximate (isotropic) treatment of cell s.u.'s is used for estimating s.u.'s involving l.s. planes.
Refinement. Refinement of F^2^ against ALL reflections. The weighted R-factor wR and goodness of fit S are based on F^2^, conventional R-factors R are based on F, with F set to zero for negative F^2^. The threshold expression of F^2^ > 2σ(F^2^) is used only for calculating R-factors(gt) etc. and is not relevant to the choice of reflections for refinement. R-factors based on F^2^ are statistically about twice as large as those based on F, and R- factors based on ALL data will be even larger.

### Fractional atomic coordinates and isotropic or equivalent isotropic displacement parameters (Å^2^)

**Table:**

	*x*	*y*	*z*	*U*_iso_*/*U*_eq_	
O1	−0.07705 (4)	1.29454 (6)	0.44060 (5)	0.01923 (12)	
O2	0.23879 (4)	0.85691 (6)	0.26656 (5)	0.01752 (11)	
O3	0.34273 (4)	0.90704 (7)	0.45979 (5)	0.02001 (12)	
C7	0.17088 (6)	1.07237 (8)	0.50635 (6)	0.01751 (13)	
C8	0.08861 (6)	1.16244 (9)	0.52300 (6)	0.01815 (13)	
C9	0.00128 (5)	1.20628 (7)	0.42014 (6)	0.01493 (12)	
C10	−0.00280 (5)	1.15864 (8)	0.30079 (6)	0.01519 (12)	
C11	0.07897 (5)	1.06554 (8)	0.28576 (6)	0.01482 (12)	
C12	0.16699 (5)	1.02150 (7)	0.38786 (6)	0.01410 (11)	
C13	0.25438 (5)	0.92210 (8)	0.37051 (6)	0.01435 (12)	
N1	0.48836 (5)	0.71343 (7)	0.42194 (5)	0.01544 (11)	
N2	0.39201 (5)	0.66496 (8)	0.21647 (6)	0.01851 (12)	
C1	0.48185 (5)	0.64504 (7)	0.31296 (6)	0.01461 (12)	
C2	0.57195 (6)	0.55617 (8)	0.30846 (6)	0.01732 (13)	
C3	0.65954 (6)	0.53911 (8)	0.41374 (7)	0.01848 (13)	
C4	0.66298 (5)	0.60787 (8)	0.52806 (6)	0.01614 (12)	
C5	0.57535 (5)	0.69542 (8)	0.52670 (6)	0.01641 (12)	
C6	0.75785 (6)	0.58625 (10)	0.64330 (7)	0.02191 (14)	
H2A	0.5718 (11)	0.5103 (16)	0.2257 (12)	0.027 (3)*	
H3A	0.7256 (12)	0.4762 (18)	0.4148 (13)	0.034 (3)*	
H5A	0.5712 (11)	0.7533 (16)	0.5988 (13)	0.027 (3)*	
H6A	0.7657 (12)	0.4804 (19)	0.6662 (14)	0.039 (4)*	
H6B	0.8244 (13)	0.6124 (18)	0.6294 (14)	0.038 (4)*	
H6C	0.7483 (13)	0.6507 (19)	0.7124 (16)	0.040 (4)*	
H7A	0.2334 (11)	1.0425 (17)	0.5794 (13)	0.029 (3)*	
H8A	0.0899 (13)	1.2010 (18)	0.6068 (15)	0.038 (4)*	
H10A	−0.0628 (12)	1.1968 (16)	0.2289 (14)	0.030 (3)*	
H11A	0.0758 (10)	1.0333 (15)	0.2012 (11)	0.021 (3)*	
H101	−0.1257 (15)	1.3172 (19)	0.3673 (16)	0.045 (4)*	
H1N1	0.4304 (13)	0.7749 (19)	0.4310 (14)	0.038 (4)*	
H1N2	0.3819 (12)	0.6231 (15)	0.1407 (13)	0.028 (3)*	
H2N2	0.3394 (11)	0.7245 (15)	0.2326 (12)	0.025 (3)*	

### Atomic displacement parameters (Å^2^)

**Table:**

	*U*^11^	*U*^22^	*U*^33^	*U*^12^	*U*^13^	*U*^23^
O1	0.0165 (2)	0.0239 (3)	0.0175 (2)	0.00637 (17)	0.00561 (18)	0.00151 (18)
O2	0.0140 (2)	0.0240 (2)	0.0143 (2)	0.00056 (16)	0.00405 (16)	−0.00257 (17)
O3	0.0149 (2)	0.0292 (3)	0.0137 (2)	0.00667 (18)	0.00134 (16)	−0.00034 (18)
C7	0.0160 (3)	0.0233 (3)	0.0122 (2)	0.0047 (2)	0.0029 (2)	0.0015 (2)
C8	0.0176 (3)	0.0240 (3)	0.0124 (2)	0.0053 (2)	0.0041 (2)	0.0011 (2)
C9	0.0131 (2)	0.0166 (3)	0.0150 (2)	0.00114 (18)	0.00441 (19)	0.00139 (19)
C10	0.0128 (2)	0.0175 (3)	0.0136 (2)	0.00088 (19)	0.00182 (19)	0.00068 (19)
C11	0.0137 (2)	0.0171 (3)	0.0126 (2)	0.00074 (19)	0.00256 (19)	0.00033 (19)
C12	0.0123 (2)	0.0169 (2)	0.0127 (2)	0.00125 (18)	0.00344 (18)	0.00125 (19)
C13	0.0125 (2)	0.0180 (3)	0.0127 (2)	0.00072 (19)	0.00415 (19)	0.00162 (19)
N1	0.0147 (2)	0.0176 (2)	0.0136 (2)	0.00132 (17)	0.00391 (18)	−0.00160 (17)
N2	0.0164 (2)	0.0227 (3)	0.0144 (2)	0.00148 (19)	0.00197 (19)	−0.00235 (19)
C1	0.0152 (2)	0.0154 (2)	0.0135 (2)	−0.00101 (18)	0.0050 (2)	−0.00051 (19)
C2	0.0187 (3)	0.0187 (3)	0.0155 (3)	0.0024 (2)	0.0067 (2)	−0.0014 (2)
C3	0.0174 (3)	0.0202 (3)	0.0185 (3)	0.0030 (2)	0.0066 (2)	−0.0001 (2)
C4	0.0144 (2)	0.0177 (3)	0.0158 (3)	−0.0001 (2)	0.0041 (2)	0.0008 (2)
C5	0.0160 (3)	0.0193 (3)	0.0134 (2)	0.0003 (2)	0.0039 (2)	−0.0015 (2)
C6	0.0163 (3)	0.0271 (3)	0.0193 (3)	0.0001 (2)	0.0013 (2)	0.0036 (2)

### Geometric parameters (Å, °)

**Table:**

O1—C9	1.3541 (8)	N1—C5	1.3636 (9)
O1—H101	0.893 (18)	N1—H1N1	0.956 (16)
O2—C13	1.2670 (8)	N2—C1	1.3331 (9)
O3—C13	1.2728 (8)	N2—H1N2	0.904 (14)
C7—C8	1.3875 (9)	N2—H2N2	0.922 (14)
C7—C12	1.4004 (9)	C1—C2	1.4187 (9)
C7—H7A	0.993 (14)	C2—C3	1.3705 (10)
C8—C9	1.3978 (9)	C2—H2A	1.020 (13)
C8—H8A	1.003 (15)	C3—C4	1.4169 (10)
C9—C10	1.4003 (9)	C3—H3A	1.016 (15)
C10—C11	1.3909 (9)	C4—C5	1.3675 (9)
C10—H10A	0.990 (15)	C4—C6	1.4962 (10)
C11—C12	1.3984 (9)	C5—H5A	0.978 (14)
C11—H11A	0.987 (12)	C6—H6A	0.963 (16)
C12—C13	1.4912 (9)	C6—H6B	0.952 (16)
N1—C1	1.3516 (8)	C6—H6C	1.006 (17)
			
C9—O1—H101	108.4 (11)	C1—N2—H1N2	123.5 (9)
C8—C7—C12	121.04 (6)	C1—N2—H2N2	114.9 (8)
C8—C7—H7A	119.8 (8)	H1N2—N2—H2N2	121.6 (12)
C12—C7—H7A	119.1 (8)	N2—C1—N1	118.50 (6)
C7—C8—C9	119.89 (6)	N2—C1—C2	123.94 (6)
C7—C8—H8A	122.5 (9)	N1—C1—C2	117.56 (6)
C9—C8—H8A	117.6 (9)	C3—C2—C1	119.59 (6)
O1—C9—C8	117.93 (6)	C3—C2—H2A	121.2 (8)
O1—C9—C10	122.31 (6)	C1—C2—H2A	119.2 (8)
C8—C9—C10	119.76 (6)	C2—C3—C4	121.81 (6)
C11—C10—C9	119.73 (6)	C2—C3—H3A	122.2 (8)
C11—C10—H10A	122.0 (9)	C4—C3—H3A	116.0 (8)
C9—C10—H10A	118.2 (9)	C5—C4—C3	116.31 (6)
C10—C11—C12	121.02 (6)	C5—C4—C6	122.13 (6)
C10—C11—H11A	119.2 (7)	C3—C4—C6	121.56 (6)
C12—C11—H11A	119.7 (7)	N1—C5—C4	122.03 (6)
C11—C12—C7	118.53 (6)	N1—C5—H5A	114.7 (8)
C11—C12—C13	120.53 (6)	C4—C5—H5A	123.2 (8)
C7—C12—C13	120.94 (6)	C4—C6—H6A	110.2 (9)
O2—C13—O3	122.08 (6)	C4—C6—H6B	111.2 (9)
O2—C13—C12	118.84 (6)	H6A—C6—H6B	104.7 (13)
O3—C13—C12	119.08 (6)	C4—C6—H6C	109.8 (9)
C1—N1—C5	122.65 (6)	H6A—C6—H6C	111.4 (13)
C1—N1—H1N1	121.6 (9)	H6B—C6—H6C	109.5 (13)
C5—N1—H1N1	115.7 (9)		
			
C12—C7—C8—C9	−1.35 (11)	C11—C12—C13—O3	166.93 (6)
C7—C8—C9—O1	−179.81 (6)	C7—C12—C13—O3	−13.29 (10)
C7—C8—C9—C10	0.17 (11)	C5—N1—C1—N2	177.71 (6)
O1—C9—C10—C11	−178.73 (6)	C5—N1—C1—C2	−2.43 (10)
C8—C9—C10—C11	1.29 (10)	N2—C1—C2—C3	−178.20 (7)
C9—C10—C11—C12	−1.60 (10)	N1—C1—C2—C3	1.94 (10)
C10—C11—C12—C7	0.44 (10)	C1—C2—C3—C4	0.08 (11)
C10—C11—C12—C13	−179.77 (6)	C2—C3—C4—C5	−1.66 (11)
C8—C7—C12—C11	1.05 (11)	C2—C3—C4—C6	178.74 (7)
C8—C7—C12—C13	−178.74 (6)	C1—N1—C5—C4	0.83 (11)
C11—C12—C13—O2	−12.12 (10)	C3—C4—C5—N1	1.25 (10)
C7—C12—C13—O2	167.67 (6)	C6—C4—C5—N1	−179.16 (6)

### Hydrogen-bond geometry (Å, °)

**Table:**

*D*—H···*A*	*D*—H	H···*A*	*D*···*A*	*D*—H···*A*
N1—H1N1···O3	0.957 (17)	1.726 (17)	2.6738 (9)	170.4 (15)
N2—H1N2···O3^i^	0.905 (14)	1.967 (14)	2.8443 (9)	162.9 (13)
N2—H2N2···O2	0.922 (14)	1.876 (14)	2.7962 (9)	176.1 (13)
O1—H101···O2^ii^	0.892 (18)	1.779 (18)	2.6635 (8)	170.7 (19)
C3—H3A···O2^iii^	1.016 (16)	2.476 (15)	3.1887 (9)	126.7 (10)

Symmetry codes: (i) *x*, −*y*+3/2, *z*−1/2; (ii) −*x*, *y*+1/2, −*z*+1/2; (iii) −*x*+1, *y*−1/2, −*z*+1/2.
